# INSL3: an informative testis biomarker for assessing the HPG axis in men

**DOI:** 10.3389/fendo.2026.1911172

**Published:** 2026-10-05

**Authors:** Ravinder Anand-Ivell, Xinyuan Yang, Bilal Tulumcu, Richard Ivell

**Affiliations:** 1 School of Bioscience, University of Nottingham, Sutton Bonington, United Kingdom; 2 School of Veterinary Medicine and Science, University of Nottingham, Sutton Bonington, United Kingdom

**Keywords:** HPG-axis, hypogonadism, hypothalamo-pituitary-gonadal axis, INSL3, Leydig cell, RXFP2, testis

## Abstract

The male hypothalamo–pituitary–gonadal (HPG) axis is an integrated endocrine system linking hypothalamic GnRH secretion, pituitary gonadotropins (LH and FSH), Leydig cell steroidogenesis, Sertoli cell function, and spermatogenesis. While conventional parameters such as testosterone or LH may be subject to high biological variance due to their pulsatile mode of secretion and multiple stress-related or environmental influences, the Leydig-cell-secreted peptide insulin-like peptide 3 (INSL3) offers a robust and constitutive alternative reflecting Leydig cell number, differentiation status, and long-term functional capacity. This narrative review considers INSL3 across the male life course in relation to other components of the HPG axis, including in fetal development, minipuberty, pubertal development, and male aging. It shows how measuring INSL3 offers important granularity in understanding issues such as the effects of environmental endocrine disruption, childhood, and lifestyle influences on pubertal development as well as on adult Leydig cell capacity, hypogonadism, and age-related morbidity. The presented evidence suggests that INSL3 will prove to be a useful biomarker for the HPG axis in adult life, though the reference range in healthy adults is quite large for yet unknown reasons; associations with BMI as well as early childhood and adolescent influences are described. While INSL3 reflects the GnRH–LH–testosterone arm of the HPG axis and is normally not directly linked to the spermatogenic compartment and male fertility, some studies do suggest a supportive relationship which merits further research. Similarly, other functions of INSL3 in men also remain underexplored.

## Introduction

Conventionally, the hypothalamo–pituitary–gonadal (HPG) axis in the male is one of several major endocrine axes which indicate how information in the brain, focused through the hypothalamus and anterior pituitary, is translated into circulating hormones, first as the gonadotropins LH and FSH in the pituitary and subsequently as sex steroids generated by the Leydig cells of the testes as well as into sperm production by the seminiferous tubules (**Figure 1**). Regulation and modulation of the HPG axis occurs at multiple levels serving to integrate peripheral information into the core modules of the axis. This occurs (a) by negative feedback of steroids (testosterone and estradiol) to the hypothalamus and pituitary and of the Sertoli cell peptide inhibin B to the pituitary and (b) by subtle regulation of the GnRH pulse generator in the hypothalamus, itself determining the specific phasic release of FSH and LH from the anterior pituitary (1, 2). The health and status of the HPG axis is generally assessed by measuring the circulating levels of the pituitary gonadotropins LH and/or FSH as well as by either semen parameters and/or testosterone as end-products. All of these parameters are subject to high technical and biological variance, leading to incomplete assessment of HPG axis health. Moreover, significantly reduced function (hypogonadism) can manifest itself as severely reduced androgen production and/or spermatogenesis, depending on which branch of the axis (LH-driven or FSH-driven) is most affected. This can be caused by dysfunction at any level of the axis, leading to the concepts of primary (testis dysfunction) or secondary (pituitary and/or hypothalamic dysfunction, also known as central) hypogonadism.

**Figure 1:**
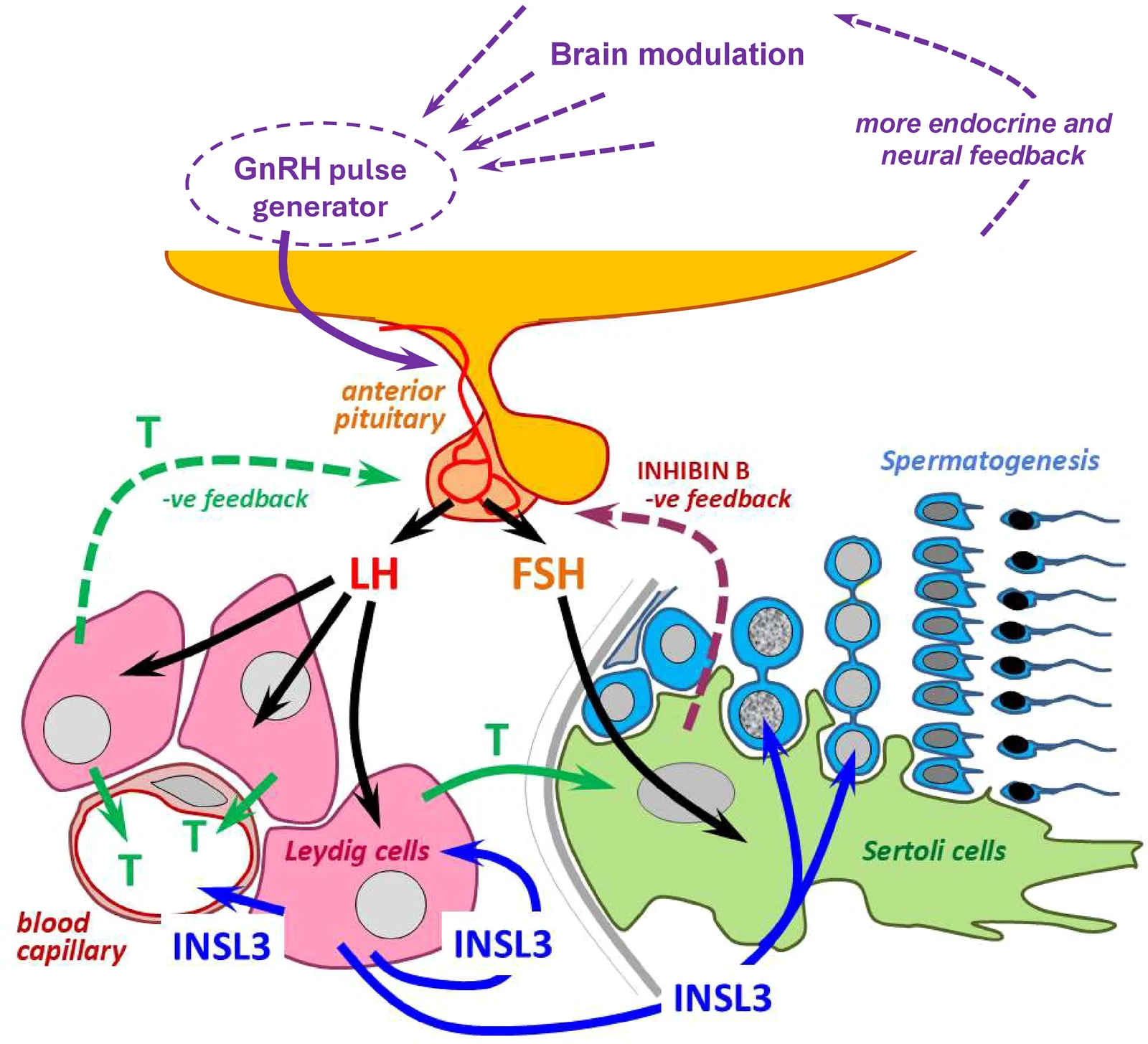
Schematic to show the components of the HPG axis, including INSL3 acting through its receptor RXFP2. Arrows indicate feedback and other regulations implicated in the modulation of the HPG axis [modified after (33)].

The discovery in the 1990s (3–5) of the peptide hormone insulin-like peptide 3 (INSL3) as a specific and constitutively secreted product of the steroidogenic Leydig cells of the testes has substantially increased the understanding of their function. While INSL3 plays an essential role in the process of testicular descent from a peri-renal position into the inguinal region during fetal life, there is accumulating evidence that INSL3 may prove to be a useful biomarker for the HPG axis in adult life. Here we aim to show that it differs from others in that it:

- specifically indicates the differentiation status and functionality of the Leydig cells and no other component of the HPG axis,
- represents only the LH branch of the HPG axis and is mostly independent of the FSH branch,
- is quantitatively very consistent (low technical and biological variance) within an individual, acting as a long-term monitor of Leydig cell functional capacity, and,
- is not secreted in a pulsatile or phasic manner like other components of the HPG axis.

The hormone INSL3 acts via its specific G-protein-coupled membrane receptor, called relaxin family peptide receptor 2 (RXFP2) (6, 7), with endocrine functions so far described in the adult for bone and skeletal muscle (8), the adrenal cortex and kidney (9, 10), and within the testes themselves (spermatocytes and Leydig cells; see **Figure 1**) (11). Within the fetus, INSL3 is responsible via RXFP2 for the first transabdominal phase of testis descent (11–13). No other known factor appears able to act through RXFP2, making this a unique hormone–receptor dyad, and so far there appears to be no known feedback mechanism involving RXFP2 and the HPG axis.

In this narrative review, we attempt to summarize what is known about INSL3 as a circulating blood parameter in men, specifically representing the functional capacity of the LH-target cells (Leydig cells) within the testes and their relationship to other parameters of the HPG axis.

## Constitutive expression

INSL3 appears to differ from many peptide and other hormones in that it appears to be secreted from Leydig cells in a constant, unregulated fashion, thereby reflecting simply the total number of Leydig cells and their differentiation status. INSL3 peptide is mostly secreted quickly and quantitatively soon after the gene has been transcribed. This constitutive expression is in contrast to the regulated expression of many hormones which can occur in cells at both transcriptional as well as translational, enzymatic, and/or secretory levels—for example, the production of steroids such as testosterone is acutely influenced within minutes or hours by secretagogues such as LH or hCG, causing a considerable acute upregulation of mRNA encoding the StAR (steroidogenic acute regulatory) protein and/or various steroidogenic enzymes and the consequent increased release of steroid from the cells into the surrounding environment (14). The constitutive expression of INSL3 is best illustrated in Leydig cell culture experiments using primary cells or secondary cell lines (**Figure 2**) where, unlike steroids such as progesterone or testosterone, INSL3 secretion is unaffected by a range of conventional secretagogues or by increasing concentrations of the LH receptor agonist hCG (15, 16).

**Figure 2:**
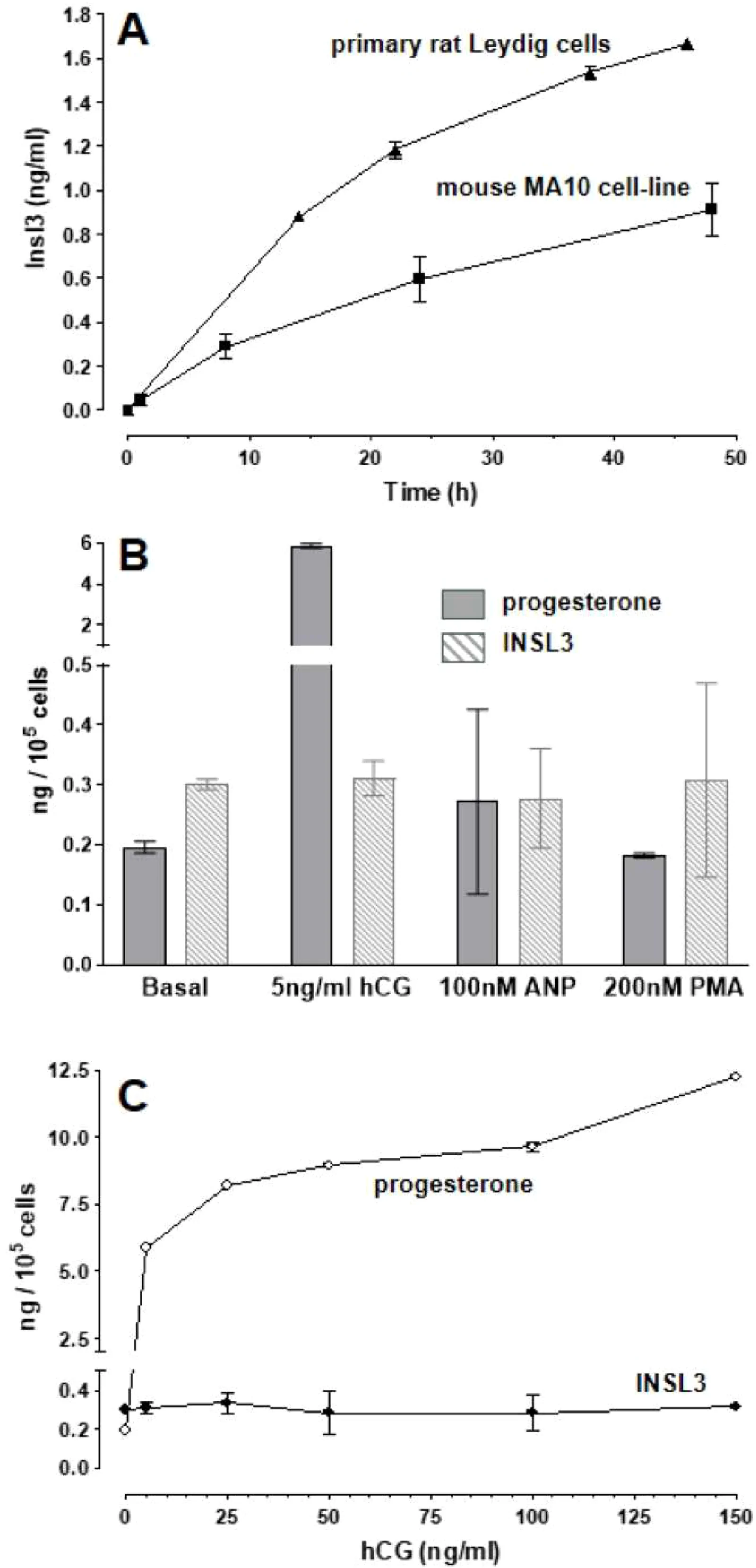
**(A)** Time course of INSL3 production from primary adult rat Leydig cells and the mouse MA10 cell line. **(B)** Influence of various Leydig cell effectors known to stimulate different signaling pathways on either cumulative INSL3 secretion (after 20 h) or progesterone secretion (after 3 h) in the same MA10 Leydig cell cultures. hCG, human chorionic gonadotropin; ANP, atrio-natriuretic peptide; PMA, phorbol myristate acetate. While for INSL3 there is no effect of any compound compared to basal, for progesterone, particularly hCG is highly stimulatory. **(C)** Dose–response curves for hCG on cultured MA10 cells, measuring either progesterone (after 3 h) or INSL3 (after 20 h), as indicated. All data represent mean ± SD [reproduced from (16)].

Similar conclusions can be drawn from studies involving the transfection of INSL3 gene promoter–reporter constructs into Leydig or other cells. Firstly, the proximal promoter region of the INSL3 gene is likely very short in all species studied, being located either within or very close to the 3’ terminal region of the JAK3 gene (11, 17). Various experiments have shown that the INSL3 promoter is constitutively activated in Leydig cells only by the orphan transcription factors Nr5A1 and Nr4A1 or influenced by related factors such as COUP-TF2 and that, generally, this cannot be affected by additional signaling pathways (15, 18). However, in supraphysiological experimental contexts, it has been shown that steroid-activated estrogen and androgen receptors, by acting on specific responsive elements in the proximal INSL3 gene promoter in a non-classical manner, may also modulate gene activity (19). In this context, it is important to note that adult Leydig cells *in vivo* will inevitably have ligand-saturated steroid receptors and will be anyway out-competed for binding to the INSL3 proximal promoter by an excess of orphan transcription factors such as Nr5A1 or Nr4A1 so that such possible steroid regulation *in vivo* is unlikely. However, since the transcription factor Nr4A1 itself can be induced in Leydig cells by cAMP (20), it is possible that in less acute contexts or in less differentiated (immature) Leydig cells LH may be able to stimulate INSL3 gene expression, though the cell culture endogenous expression systems (**Figure 2**) suggest that longer periods than 48 h might be needed to achieve an effect. *In vivo* studies during puberty or its equivalent following ethane dimethane sulfonate (EDS) treatment in rats (21) showed that the increasing level of INSL3 at this time was primarily due to the proliferation of immature Leydig cell stages (see later). In humans, once terminal differentiation status of Leydig cells is achieved at around 20–24 years, INSL3 secretion by these cells appears to be no longer acutely influenced even by high concentrations of LH or hCG (22).

The constitutive expression outlined above relates to stable Leydig cell lines or to primary Leydig cells which have achieved their mature, end-differentiated status. The situation may be different for less differentiated Leydig precursor cells or other situations *in vivo* where the testicular environment—for example, testicular blood flow—may be influenced by phasic hormones such as testosterone and/or LH (23–25). The latter may explain a small diurnal variation in circulating INSL3 observed in normal adult men paralleling the much larger phasic secretion of testosterone (26), though this has not been observed by others (27). In young adult rats and humans, once peak INSL3 is attained at the end of puberty, circulating levels decline to a lower steady-state in young adulthood (16, 22, 26, 28), suggesting that some of the Leydig cells may have partially dedifferentiated as the pubertal LH storm subsides. There is no evidence of any Leydig cell loss at this time. This might then explain why INSL3 levels in some normal men can increase by some 27% 3 days after stimulation with 5,000 U hCG (26). Contrarily, suppression of the HPG axis following administration of cyproterone acetate and testosterone to normal men for 16 weeks can lead to a reduction of INSL3 (29); some LH is required to maintain Leydig cell differentiation status. In young adult male goats, it was reported that INSL3 may also be secreted within 1 h of GnRH stimulation, though to a much lesser extent than for testosterone, and can be suppressed by a GnRH antagonist (30). All of these *in vivo* observations are relatively slow and likely represent chronic changes to Leydig cell differentiation status and/or testicular blood flow rather than the acute changes seen with conventional regulated secretion *in vitro*, nor do they preclude other indirect effects due to the concomitant large increases in testosterone.

In rodents and primates, including humans, Leydig cells undergo at least two discrete phases of differentiation (31): in the fetus from stem cells in the fetal testes sharing ontogeny with similar adrenocortical cells and attaining a mature morphology in the second trimester (human) or at term (rodents) and during puberty with, in higher primates, an additional third short phase of development in the first postnatal months, referred to as “mini-puberty” (32). In all phases, there is a *de novo* differentiation of Leydig cells from Leydig stem cells (LSC) or progenitor cells, through immature Leydig cell (ILC) stages, to mature adult-type Leydig cells (ALC). The proliferation and differentiation of the less mature stages, as well as the maintenance of the mature Leydig cell phenotype, appear to be under the influence of LH (or hCG) acting through LH receptors. This is well illustrated by the experiments shown in **Figure 3** where relatively undifferentiated ILC prepared from the testes of postnatal day 10 rats are subject *in vitro* to hCG stimulation for up to 2 weeks. Unlike what is observed with the MA-10 mouse Leydig cell line (**Figure 2**), the immature rat Leydig cells are stimulated to both differentiate and proliferate with a consequent massive increase in INSL3 secretion (33). A comparable chronic increase in circulating INSL3 can be observed in hypogonadotropic hypogonadal male infertility patients treated with hCG over weeks rather than only a few days (29).

**Figure 3:**
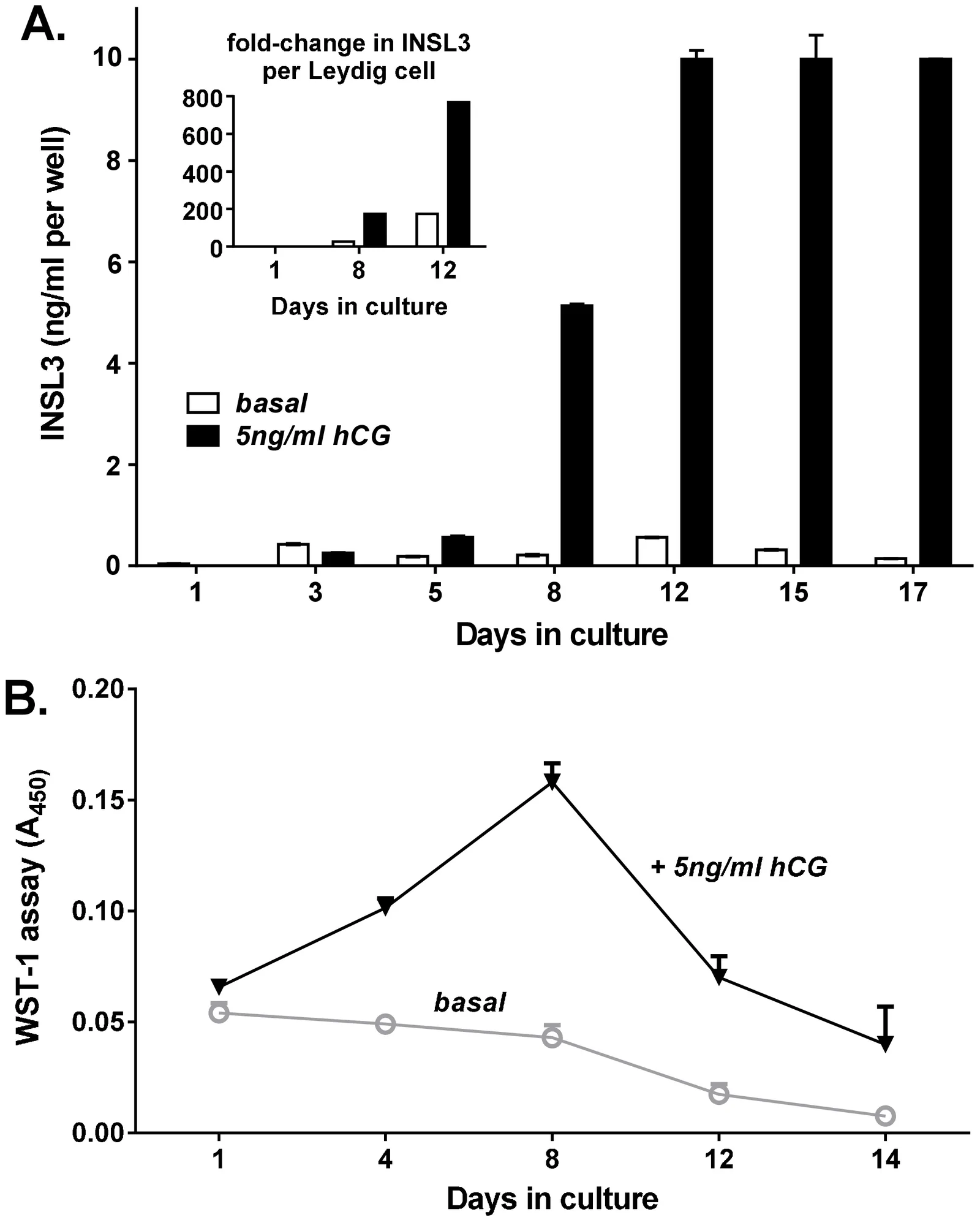
**(A)** Long-term culture of purified primary Leydig cells prepared from postnatal day (PND) 10 rats with or without stimulation by hCG as indicated. The inset is similar data, now taking account of differential cell proliferation [see **(B)**]. **(B)** Change in cell density of similar PND10 Leydig cell cultures as in **(A)** assessed using the WST-1 cell proliferation assay. All data represent mean ± SD [reproduced from (33)].

Therefore, when measuring and interpreting Leydig cell INSL3 in blood or other body fluids or in cell culture media, it is essential to consider whether the Leydig cells from which the peptide hormone derives are in a mature steady-state (i.e., terminally or fully differentiated) or in an immature state capable of being further differentiated or may have lost their differentiation status (dedifferentiation). Why this is important will be considered in the following sections.

## INSL3 in the fetus and the impact of endocrine disruption on early Leydig stem and precursor cells

INSL3 is first produced during fetal life at around gestational week 10 (time after last menses or approximately week 8 post-coitum, pc) in humans or gestational day 13 pc in mice and appears to accompany the differentiation of the fetal Leydig cell population from stem cells (34). INSL3 is only detectable in male fetuses, with no expression at all within female fetuses (35). A recent study in mouse using the natural INSL3 gene promoter to drive the expression of a TdTomato fluorophore *in vivo* also confirmed no expression on day 12.5 and first expression on day 13 uniquely within the testicular interstitial cells and no other cell type in the body (36). All evidence suggest that INSL3 peptide is made almost immediately after the first appearance of INSL3 mRNA (34, 37), again implying a constitutive mode of secretion. In the mothers of human male fetuses, INSL3 can be measured in amniotic fluid already at gestational week 11, though it increases to a maximum at between weeks 12 to 16 before declining in the second half of pregnancy (37). This pattern reflects well the functional role of fetal INSL3 to promote the first transabdominal phase of testicular descent whereby INSL3 acts on RXFP2 receptors in the gubernacular ligaments linking the testes to the inguinal region. These expand and anchor the testes, retaining them close to the inguinal body wall prior to the second phase of descent when the testes migrate into the scrotum.

In other species, such as rodents, pig, or bovine, the first phase of testis descent may be prolonged into the perinatal or even postnatal phase, with fetal INSL3 remaining at high levels until birth or shortly thereafter (38). In most species, fetal Leydig cell differentiation and proliferation are driven by pituitary LH. Mice appear to be an exception since this role is filled by ACTH at least in early gestation (39) and possibly also by PACAP (40), which may be why *hpg* mice with a genetically disrupted HPG axis, lacking GnRH, show an apparently normal population of fetal Leydig cells at birth, with normal testis location in the scrotum and hence normal INSL3 expression in fetal Leydig cells, though not as adults (41). Moreover, in humans and higher primates, trophoblast hCG may also contribute to driving Leydig cell morphogenesis, though it may be dispensable in early pregnancy (42). Both LH/hCG and ACTH—even CRF—act on their respective membrane receptors to activate cAMP-driven pathways.

Although in the human fetus INSL3 appears to decline toward term of pregnancy, it is still measurable in cord blood at birth, varying in control subjects from being undetectable to 0.4 ng/mL (43–45), compared to 0.4 to 2.0 ng/mL in fetal venous blood at gestational weeks 15–20 (46). Importantly, several studies have shown that INSL3 concentrations in human cord blood at term may be significantly reduced if the mothers show evidence of exposure to environmental endocrine-disrupting agents such as phthalates (44). The largest study to assess the impact of phthalate exposure on the human fetus made use of INSL3 measurement of amniotic fluid samples collected at amniocentesis (approximately weeks 12 to 20), where they showed a negative relationship between the concentration of phthalate or PFOS metabolites and INSL3 concentration (47, 48). Interestingly, INSL3 concentration appeared to be increased in cryptorchid cases (37). While this appears counter-intuitive given the role of INSL3 in testicular descent, the results rather suggest a disruption of the dynamics of fetal Leydig cell differentiation, with cryptorchidism being associated with both an earlier peak of amniotic INSL3 concentration and an increased variance (37).

Such studies in humans are scarce. However, INSL3 has become increasingly important as a biomarker to monitor endocrine disruption in animal models such as rats. Maternal exposure to xenoestrogens or phthalates leads to a reduced development of the fetal Leydig cell population and consequently to a lower INSL3 expression (49, 50). Findings were similar using human fetal testicular explants exposed to phthalates as well as to common analgesics such as paracetamol (acetaminophen) or ibuprofen (51, 52) that can cross the placenta and adversely affect the fetus.

A close inspection of all such studies illuminates an important feature. Unlike the action of other reprotoxic environmental disrupting substances, those listed above and, in particular, phthalates do not appear to act acutely as alternative steroid receptor ligands but rather achieve their impact by otherwise altering the rates of differentiation and/or proliferation of immature Leydig cells or their stem cells. This is illustrated in **Figure 4** where adult rats were treated with a single bolus injection (on experimental day 1) of ethane dimethane sulfonate (EDS) to eliminate all mature Leydig cells. In this model, new Leydig cells redifferentiate from resident Leydig stem cells with a kinetic similar to that of normal puberty (21). The lack of Leydig cells and hence testosterone feedback to the hypothalamus and pituitary in this model leads to a supraphysiological LH drive. Brief early exposure to the endocrine disruptors dibutyl phthalate (DBP) or the xenoestrogen diethylstilbestrol (DES) on days 5 to 7 after EDS treatment both accelerated the rate of later Leydig cell proliferation and differentiation compared with controls, which were administered the vehicle only (21). Importantly, we can see that on day 27, halfway through this quasi-pubertal recovery after EDS treatment, the increased circulating INSL3 (**Figure 4D**) and its specific mRNA (**Figure 4C**) closely reflect the increased proliferation of immature Leydig cells (**Figure 4B**), whereas the testes themselves barely change weight (**Figure 4A**). Unlike circulating INSL3, the testosterone levels (**Figure 4E**) do not reflect Leydig cell numbers and, though still reduced, are not significantly different from the pre-treatment level (1.44 ± 0.33 ng/mL; mean ± SEM) (21). This model recapitulates what is observed when young male rats going through puberty have been briefly maternally exposed to either DBP or DES at the end of gestation or via lactation so that only resident Leydig stem cells can be acutely affected; there is a similar distorted pattern of INSL3 secretion during puberty (53).

**Figure 4:**
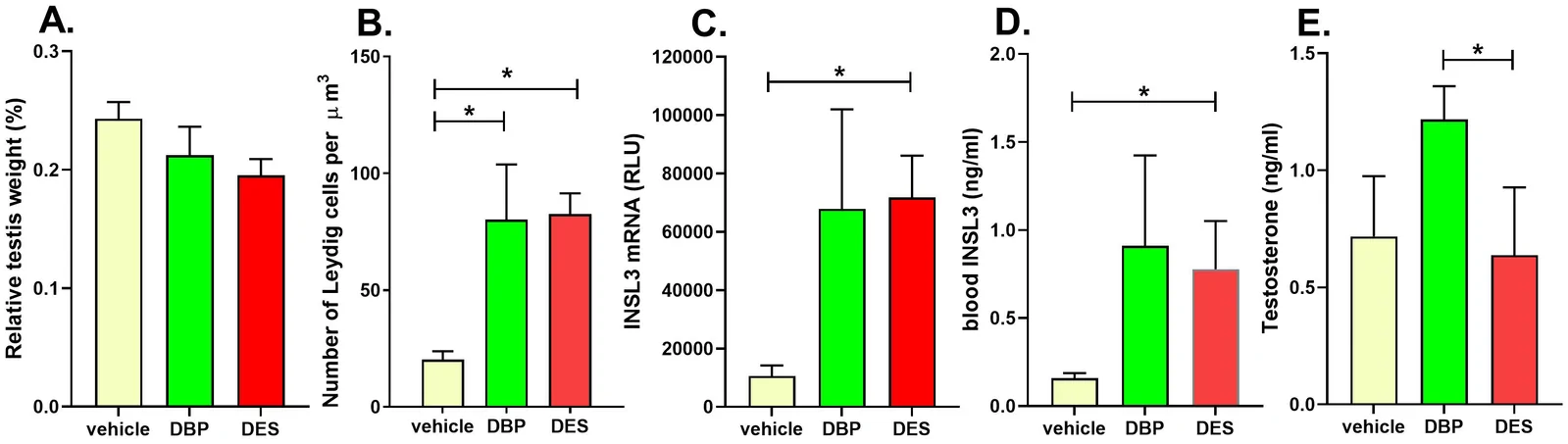
Analysis on day 27 of testes from adult (>60 days) rats treated with ethane dimethane sulfonate (EDS) on day 1 to eliminate all mature Leydig cells, followed by either vehicle, dibutyl phthalate (DBP), or diethylstilbestrol (DES) on days 5–7, as indicated. **(A)** Relative testis weight (testis weight divided by body weight). **(B)** Leydig cell numbers determined stereoscopically by the physical dissector method. **(C)** Normalized INSL3 mRNA measured by quantitative RT-PCR. **(D)** Circulating INSL3 in peripheral venous blood obtained from tail bleeds. **(E)** Circulating testosterone in peripheral venous blood obtained from tail bleeds. All data are means ± SEM (for *n* = 4 animals); asterisks indicate differences significant at *p* < 0.05 (ANOVA with Kruskal–Wallis multiple comparison) [modified after (21); q.v. for full experimental details].

In summary, exposure of Leydig stem cells or early immature stages to various endocrine-disrupting agents or certain pharmaceuticals appears to have long-lasting impacts on subsequent Leydig cell differentiation and proliferation. The extent to which this is a direct effect on these Leydig cell progenitors with evident epigenetic consequences or whether there are effects acting via other components of the HPG axis is still unclear. As a biomarker, however, circulating INSL3 appears to offer a read-out equivalent to actual Leydig cell numbers and/or their differentiation status, unlike testosterone, which is continually being compensated by pituitary LH influenced by acute homeostatic feedback.

## INSL3 in mini-puberty

Minipuberty is the period of 1 to 6 months immediately following normal birth in the human infant when there appears to be a small revival of the hormones of the HPG axis (32, 54). These hormones seem to be necessary in boys for confirming some aspects of the masculine phenotype. Detailed inspection of these hormone levels suggests that mostly those involving Sertoli cell development are emphasized, with AMH and Inhibin B having a more pronounced peak at around 3 months of age, whereas both LH and INSL3 appear only to decline soon after birth with little significant difference from values obtained from cord blood at term (54, 55). From these results, it does not appear that INSL3 is markedly involved in mini-puberty, though levels in fetal blood immediately prior to birth are not known. Peak testosterone seems slightly delayed at around 1 month of age, possibly due to a requirement for conversion from androstenedione by infant Sertoli cells. Interestingly, INSL3 during minipuberty appears to be mostly unrelated to contemporary urinary phthalate concentrations, possibly even suggesting a slight positive relationship (56). Nevertheless, a recent analysis has shown a negative association between minipuberty INSL3 concentration and hypospadias, presumably due to a general Leydig cell insufficiency and/or hypoandrogenemia associated with this condition (57). It is notable that in a regression analysis of all hormones linked to minipuberty, INSL3 showed a significant association only with calculated free testosterone (cFT) as well as LH, and with no other parameter, including total testosterone itself (55). The Leydig cells in this early postnatal phase likely represent a revived subgroup of the fetal Leydig cell population rather than a new population of Leydig cells independently differentiated from stem or precursor cells, though more research is required to clarify this point.

At least in rodents and possibly also in humans, early fetal Leydig cells appear to be gonadotropin-independent, unlike adult-type Leydig cells (42). Then, during fetal life in humans, placental hCG is believed to act on these fetal Leydig cells via the LH receptor. At mini-puberty, the surviving Leydig cells now become exclusively dependent on pituitary-derived LH. This postnatal switch from maternal hCG dependence to fetal/neonatal pituitary activation is crucial for the neonate to lay down the foundation of future spermatogenesis. Subsequently, from 6 months of age until the onset of puberty at around 8 to 9 years, hypothalamic GnRH secretion is drastically reduced (hypothalamic quiescence) for largely unknown reasons. In severe congenital hypogonadotropic hypogonadism (CHH) cases, early GnRH secretion is impaired, with infant boys presenting micropenis and testicular immaturity (58).

## INSL3 during puberty

For most mammals, a second independent generation of Leydig cells begins pubertal development immediately following birth; in rodents, this population may coexist with remnants of the fetal Leydig cell population in the first postnatal days, where the FLC can be identified by their specific expression of 17βHSD type 10 (59). In contrast, in humans, following minipuberty (see above), there is a period of hormonal quiescence during infancy and childhood until approximately 8 to 9 years of age, when the resident Leydig stem/precursor cells within the testes begin to differentiate and proliferate under the increasing governance of LH surges. This transition is accompanied by the progressive appearance of steroidogenic Leydig cell markers such as CYP11A1, CYP17A1, HSD3B, HSD17B3, and LHCGR, indicating entry into the adult Leydig cell lineage. Consistent with this, Guo and colleagues (60) showed that CYP11A1 protein expression emerges progressively during human puberty and was clearly evident only in the 14-year-old validation sample. Circulating INSL3 then provides a systemic readout of the expansion and functional maturation of this Leydig cell population.

The increasing concentration of INSL3 in circulating blood reflects this pubertal development (61–63), acting very much as a biochemical Tanner staging, with high between-individual variation. At the population level, a 10-fold variance is observed between boys at the end of puberty, presumably due to differing times of pubertal onset as well as variability in Leydig cell proliferation and their differentiation stage (64, 65). Whereas the final Tanner stage 5, based on secondary sexual characteristics, is attained at around 15 years, the INSL3 levels continue to increase, suggesting that the end of puberty, corresponding to the attainment of terminally differentiated Leydig cells and to their final functional capacity, is not reached until approximately 22 years of age (65). Tanner staging relies on the development of an androgenic phenotype, i.e., mostly reflecting testosterone production, itself acutely regulated by feedback to the hypothalamus and pituitary and subject to high pulsatility and phasic expression. At 15 years, there is more than sufficient testosterone produced to activate androgen receptors in target tissues. INSL3 secretion, however, continues to increase as it appears not to be subject to such acute negative feedback regulation of the HPG axis. After reaching maximal INSL3 concentration in the early 20s, the circulating INSL3 levels begin to decline to a relatively stable level in young adulthood at around 24 years (65). Presumably, this decline reflects the final or terminal differentiation of Leydig cells, while some Leydig cells may either die or more likely remain only partially differentiated (33). Very similar pubertal trajectories of circulating INSL3 are also seen in other mammal species, such as rat (16) and bovine (66). This differs from the profile for total testosterone which maintains its peak level into adulthood, homeostatically regulated by acute steroid feedback to the brain and pituitary (33). Hence, the serum INSL3 levels indeed seem to reflect the true functional capacity of Leydig cells in both testes, i.e., equivalent to the product of Leydig cell numbers and their average differentiation status.

Within any one individual, the adult Leydig cell population appears to be maintained with little variation across the lifespan and only minimal attrition due to Leydig cell death or replacement (see later); this is reflected in the longitudinal stability of INSL3 within adult men, for example, its consistency when measured in individuals several years apart (67, 68). Importantly, this Leydig cell functional capacity in young adulthood, as indicated by circulating INSL3, exhibits high variability *between* individuals, with values in the normal healthy population varying by more than 10-fold (67, 69), suggesting that already at this early age there are men whose INSL3 level (INSL3 <0.4 ng/mL (68); see later) would allow one to speculate that they might develop hypogonadism. Therefore, whatever determines the final Leydig cell functional capacity in young adulthood is likely to be responsible for the important androgenic role of the testes with regard to later health and morbidity (see later). However, not a lot is known about the factors which regulate the rates of pubertal proliferation and differentiation of the adult Leydig cell population and, hence, what determines the final Leydig cell functional capacity in the young adult and the risk of later hypogonadism.

This question was addressed by exploiting the large Avon Longitudinal Study of Parents and Children (ALSPAC) cohort. Young adult INSL3 was measured at 24 years of age in approximately 1,200 young men and correlated with a range of parameters from the same individuals over their preceding lifetime back to infancy as well as including some maternal factors (28, 65). Besides contemporary associations with being overweight in late puberty, the most significant associations were with being overweight in early puberty and infancy as well as having suffered viral infections such as chickenpox in early childhood (65). A relationship was also identified between young adult INSL3 concentration and prepubertal Vitamin D, though no other nutritional factors appeared to be involved (70). No clear associations were observed with the maternal parameters examined nor with pubertal timing or growth. Significantly, the results appeared to highlight as sensitive the period in childhood before puberty onset at around 8 to 9 years of age, suggesting that the early resident Leydig stem and precursor cells at this time were most vulnerable to negative influences. A similar conclusion was drawn from an earlier study in bulls, where it was shown that the feeding regimen up to approximately 4 months of age was most decisive in determining the later pubertal trajectory of INSL3 increase (66).

Clinically, INSL3 is reduced in boys with delayed puberty and seems to distinguish constitutional delay of growth and puberty (CDGP) from congenital hypogonadotropic hypogonadism (CHH), though possibly not as well as inhibin B or AMH (71, 72). It appears that, in these conditions, Sertoli cell biomarkers may offer more discrimination than a Leydig cell biomarker like INSL3. In adults with CHH, as well as in Klinefelter syndrome, INSL3 is a good discriminant (29, 71, 73) and in the latter does not seem to respond acutely to an hCG stimulus (73). As might be anticipated, chronic treatment with testosterone has no effect on INSL3 in such cases, though long-term FSH/hCG treatment significantly elevates the circulating INSL3 levels, presumably by inducing Leydig cell differentiation and/or proliferation (74).

## INSL3 and the aging male

Since it is continually compensated by LH via homeostatic steroid feedback to the brain and pituitary in healthy men, there is a relatively small decline in circulating testosterone with increasing age (ca. 8% per decade) (75). This decrease is also offset by the age-dependent rise in SHBG, leading to a decrease in free testosterone levels (76). This is different for INSL3 which declines faster at ca. 14% per decade (67, 69) and which being constitutive is not acutely regulated by the HPG axis and, therefore, not compensated despite increasing levels of circulating LH. Similar findings were reported by Chong and colleagues (77). The decline in INSL3 levels reflects the real decline in Leydig cell functional capacity. This loss of capacity may be partly due to a small loss of Leydig cells as well as to an apparent developmental regression, with the Leydig cells of older testes tending to be smaller and less differentiated, accompanied by increasing oxidative stress, inflammation, and sclerotization of the testes (78), which were also clearly shown in the aging rat testis (79). Nevertheless, in cell culture, such cells may generate sufficient levels of testosterone (80). Several studies have estimated absolute numbers of Leydig cells in human testes relative to age, applying more or less rigorous “dissector” stereology (81). Most (80, 82, 83), though not all (84), studies do suggest a small reduction in the numbers of Leydig cells with advancing age.

As mentioned earlier, there is a large (>10-fold) variation in INSL3 between individual men which persists into older age (67, 69). This is associated with being overweight and also with multiple morbidities. In fact, within the European Male Aging Study (EMAS) cohort of community-dwelling men, INSL3 correlated better with sexual symptoms, cardiovascular disease, hypertension, diabetes, or bone density than total testosterone, largely due to the higher biological and technical variance of the latter parameter, and appeared similar in this regard to cFT (85). Using sexual symptoms as a monitor for typical late-onset hypogonadism (68), a threshold value of 0.4 ng/mL INSL3 was shown to be a better discriminator of functional hypogonadism than either total testosterone, cFT, or LH, with fewer false positives or false negatives (68). This value of 0.4 ng/mL INSL3 also represents the lower reference limit (95% confidence interval) for community-dwelling European men (aged 40 to 79 years) based on the EMAS cohort (68); a similar lower reference limit was also independently determined for a cohort of similarly aged and largely Caucasian New Zealand men using a different assay (77).

## Impact of steroid treatments, illness, and inflammation

Another area where INSL3 measurement has proved very useful is in the assessment of testicular function following steroidal interventions. While it is well established that testosterone therapy, steroidal contraception paradigms, or anabolic steroid misuse can negatively affect fertility and spermatogenesis, the impact on other aspects of testis physiology can only be assessed by measuring Leydig cell functional capacity via circulating INSL3 (29, 86, 87). In these situations, there is a significant decline in INSL3 due to the chronic suppression of the HPG axis and consequently decreased LH. This long-term loss of the LH drive, in turn, leads to loss of Leydig cell functional capacity presumably through dedifferentiation, as in seasonally breeding mammals such as deer (88). Importantly, INSL3 can monitor the quantitative extent of HPG axis suppression; this has proved useful, for example, in pigs, monitoring the establishment and persistence of contraception following an immune-based approach using GnRH as an immunogen (89). Moreover, INSL3 can also monitor the recovery from such suppression of the HPG axis, for example, in dogs (90) or in men recovering from anabolic steroid misuse (87). For the latter, it was important to note that, although circulating testosterone quickly returned to normal levels, persistently reduced INSL3 showed that Leydig cell functional capacity may have been more permanently disrupted by steroid abuse (87). It needs to be emphasized that the recovery of INSL3 after long-term suppression of the HPG axis can only occur via regeneration and redifferentiation of the Leydig cell population from resident stem cells and/or precursor cells. The capacity for recovery may, therefore, vary with age and the pool of available Leydig stem cells in the testicular interstitium.

Unlike non-dividing adult Leydig cells, prepubertal Leydig cells which may be proliferating and/or differentiating may be more sensitive to specific cancer treatments, as suggested by later elevated gonadotropin levels (91) as well as reduced INSL3 (92). High-dose radiation (>20 Gy) and gonadotoxic chemotherapy (alkylating agents like cyclophosphamide or busulfan) can destroy Leydig stem cells, leading to delayed puberty and hypogonadism (93). While much focus is on fertility preservation, hypogonadal status for life exposes them also to the risk of early and multiple comorbidities.

Hypogonadism, with reduced circulating testosterone and also INSL3, is associated with a range of morbidities in aging men, for example, diabetes, and particularly with being overweight or obese (68, 85). What is still unclear is whether the morbidity is dictating Leydig cell functionality, possibly via a systemic inflammatory situation or possibly through increasing the aromatization of testosterone by adipose tissue, or whether reduced Leydig cell functionality is itself contributing to an increased risk of morbidity and disease. The latter is supported by the positive effects of testosterone therapy on typical age-related morbidities in some men exhibiting hypogonadism (94) or by the ability of INSL3 to predict morbidity in the EMAS cohort (85). The former argument is evidenced by a recent study on men with severe COVID-19 symptoms, many of whom were hypogonadal with low INSL3 but whose INSL3 and, hence, Leydig cell functional capacity recovered into the normal range after appropriate treatment (95). Both arguments are likely to be valid, with illness and Leydig cell insufficiency (hypogonadism) exacerbating each other in a spiral of increasing morbidity.

## Relationship between INSL3 and other parameters of the HPG axis, including male fertility

Conventionally, the HPG axis in men has been assessed by measuring circulating levels of testosterone (T) or its derivative, free testosterone (FT), or sometimes also by measuring LH, the T/LH ratio, FSH, or the testicular products AMH or inhibin B. Each of these parameters has advantages and disadvantages. T and its derivatives, as well as the gonadotropins, are acutely variable, with peripheral levels changing in the course of 24 h as well as from day to day and are also influenced by metabolic stress and other environmental factors, leading to a general guideline recommendation for blood sampling, with 8 h of fasting, to be between 7:00 and 10:00 a.m., after a good night’s sleep, and repeated on at least two separate occasions that are several days apart (96). This biological variance is exacerbated by variation in assay systems (technical variance), with LC–MS/MS being considered the “gold standard” and only a few immunoassays recognized as adequate, though still with considerable laboratory-to-laboratory variation. Although there have been recent international efforts to normalize testosterone measurement (97), the result of this variance is the setting of very strict cutoff levels determining what might be considered as clinical hypogonadism, restricting the diagnosis to only a very small proportion (ca. 2%) of the adult male population (98), even though the diagnosis and associated symptoms might be affecting a much larger proportion of men. FT, either calculated from circulating albumin and SHBG concentrations (cFT) or measured by equilibrium dialysis (eFT), offers a more robust parameter, largely by combining parameters to reduce biological and technical variance. However, certain medications and underlying disease conditions can dramatically affect SHBG and albumin levels (99), which, in turn, alter the amount of free, bioavailable testosterone circulating in the blood. This can lead to men being incorrectly diagnosed as hypogonadal. Both inhibin B and AMH are less often measured and generally restricted to individuals exhibiting severe hypogonadism as children or young adults. Both represent acute feedback pathways from the testes (particularly Sertoli cells) to the brain and pituitary and appear to be most informative during early development (100, 101).

INSL3 differs from these parameters in being exclusively secreted by Leydig cells of the testes, and being constitutive shows little of the biological variance illustrated by T or LH. Moreover, the well-validated assays being used to measure INSL3 in men show limited technical variance compared to T (68). Consequently, only a single blood test, sampled at any time of the day, is sufficient to provide an accurate measure of gonadal status. The EMAS cohort allowed a direct comparison of INSL3 with T, cFT, LH, and the T/LH ratio in this population of community-dwelling aging men (67, 85). Firstly, there is only modest correlation between the parameters reflecting either general parameter variance or other factors, such as age or BMI, differentially influencing them (67). In agreement with Chong and colleagues (77), we consider INSL3 to represent an independent testis parameter. Secondly, whereas all parameters other than low testosterone appear to be significantly associated with most age-related morbidities, INSL3, like cFT, appears better able to predict a range of different morbidities several years in advance (**Figure 5** (85);). In this diagram, odds ratios were calculated from the 95% confidence intervals from the multivariate binomial logistic regressions of hormone parameter in phase 1 of the study against incidence of a particular morbidity characteristic from phase 2 of the study expressed as a categorical variable, mean 4.3 years later (84). Red horizontal bars (means ± 95% CI) indicate a statistically significant difference from the null hypothesis (=1.0). Reduced sexual function is more closely associated with low FT than T (102, 103) so that the former is considered to be a more useful indicator of functional hypogonadism (104). Interestingly, an INSL3 cutoff of 0.4 ng/mL appears to be more informative than either T or cFT for defining reduced sexual function, with fewer false positives and false negatives (68), suggesting that INSL3—all results considered together—may offer a more reliable discriminator for defining functional and organic hypogonadism.

**Figure 5:**
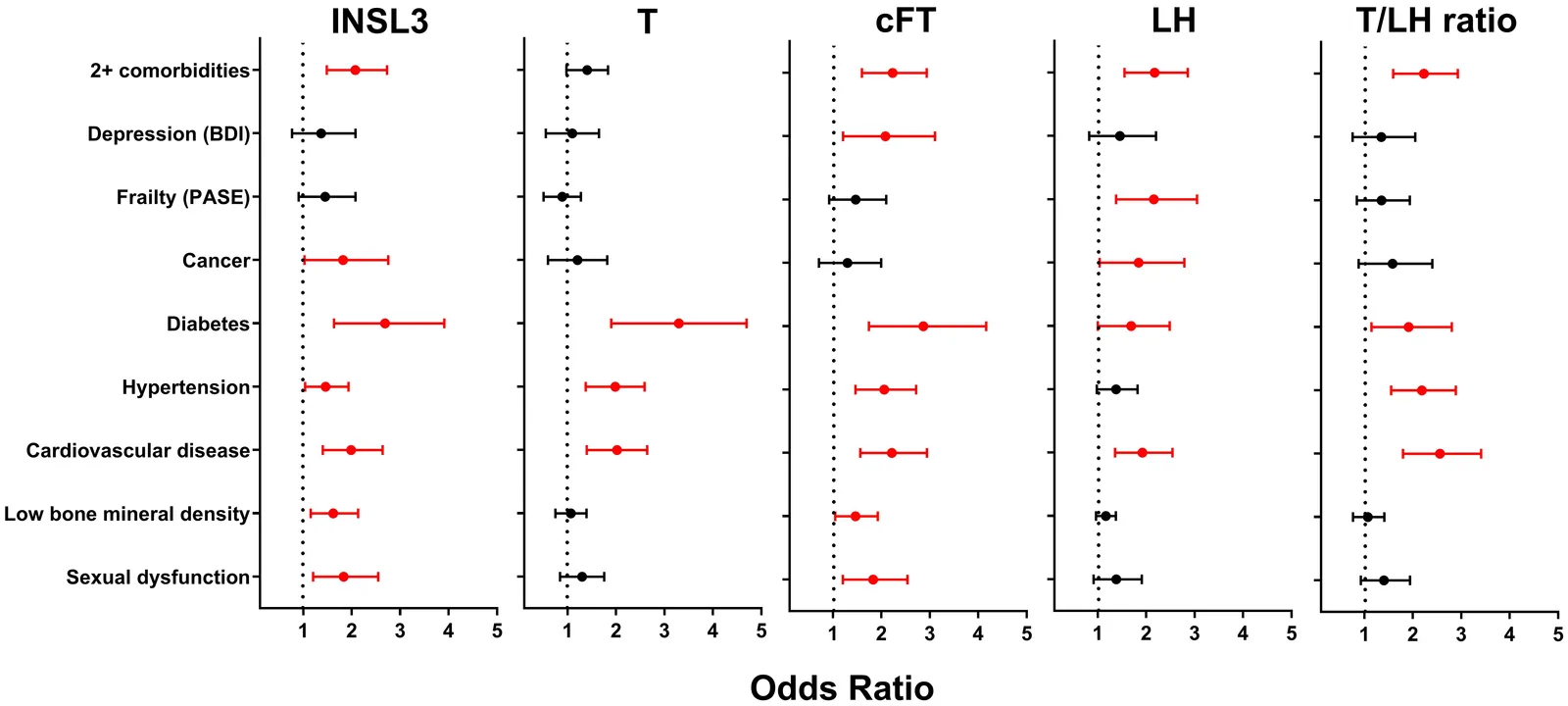
Comparative odds ratios for INSL3, total testosterone (T), calculated free testosterone (cFT), luteinizing hormone (LH), and the T/LH ratio as parameters to predict the incidence of various morbidities, as indicated, 4 to 5 years in advance. The parameters were measured in the first phase of the European Male Aging Study (EMAS) and associated with morbidity incidence for the same individuals in the second EMAS phase. Odds ratios were calculated from the 95% confidence intervals from the multivariate binomial logistic regressions of hormone parameter in phase 1 of the study against incidence of a particular morbidity characteristic from phase 2 of the study as a categorical variable mean 4.3 years later (84). Red horizontal bars (means ± 95% CI) indicate statistically significant difference from the null hypothesis (=1.0). Data represent the risk of reduced INSL3, T, cFT, the T/LH ratio, and of increased LH to be predictive of morbidity. BDI, Beckton Depression Index; sexual dysfunction was estimated from questionnaires (reproduced from (84); q.v. for full experimental and statistical details).

Although INSL3 correlates with other parameters of the HPG axis, as a uniquely Leydig cell product, it does not appear to directly reflect the status of the seminiferous compartment of the testes. In the normal male population, there is no direct association between INSL3 and conventional semen parameters (sperm number and density, volume, motility, and DNA fragmentation) (64), though interestingly it does correlate with both inhibin B and FSH in this study, which includes 300 military conscripts (age 18.2 ± 0.4 years) as well as men attending an infertility clinic (56, 64). Nor was there any significant association between INSL3 and either LH or the T/LH ratio in this study of mostly healthy younger men (64). Elsewhere other studies have suggested either a positive correlation between INSL3 and LH in young (age 29.4 ± 3.4 years) men, though not with FSH (29), or a negative relationship where the men are mostly much older and both LH and FSH are homeostatically elevated (67, 69). In these studies of aging men, the T/LH ratio correlates with INSL3 (67, 69). This contrasts with other studies suggesting a relationship between Leydig cells, circulating testosterone, and semen parameters (105–107); though in these examples, unlike the previous ones, the men were diagnosed as infertile and thus likely subject to disruption of the HPG axis affecting both LH- and FSH-dependent branches.

These findings need to be considered separately from more extreme situations such as following a steroidal contraceptive regime, where circulating INSL3 was shown to be positively associated with the level of residual spermatogenesis (108). In animal experiments, rats subjected to testicular injection of an INSL3 antagonist showed significantly reduced spermatogenesis (109), as was also observed in boars passively immunized against INSL3 (110). The specific INSL3 receptor, RXFP2, is expressed both by spermatocytes and spermatids (**Figure 1**), though not by Sertoli cells, as well as by Leydig cells themselves (111) so that INSL3 could influence sperm production either by directly acting on the seminiferous epithelium or indirectly via Leydig cells, for example, via locally increased steroidogenesis (112). Further studies are needed to characterize the role of INSL3 in infertile men.

## INSL3 in clinical practice

To date, only three INSL3 assays have achieved consistent validation against a range of reliable negative and positive samples (68). Only one of these, the Phoenix assay, is commercially available either in EIA or RIA format (Phoenix Pharmaceuticals, Burlingame, CA, USA; current detection limit 160 pg/mL (EIA) or 60 pg/mL (RIA)). Whereas earlier applications of this assay [e.g., 29], prior to around 2015, consistently suggested markedly lower population means, after that date these were more in line with the findings of the other assays (68). Both time-resolved fluorescent immunoassay (TRFIA) (7, 69) [current detection limit 20 pg/mL; with a more sensitive protocol 10 pg/mL (113)] and LC–MS/MS assays (26, 114) [current detection limit 30 pg/mL; with a more sensitive protocol 10 pg/mL (115)] are in-house assays, based respectively in Nottingham, UK, and Copenhagen, Denmark, and are used collaboratively. A joint study showed that they give essentially similar results (114). For the community-dwelling aging men of the EMAS cohort, a threshold of 0.4 ng/mL INSL3 (representing the lower 95% confidence interval, 2 SD from the population mean) was considered indicative of hypogonadism (68) (see also **Figure 6**). Whereas for younger men (19–40 years of age) the LC–MS/MS method offers a mean of 1.3 ng/mL (95% CI, 0.9–2.7) (26), the TRFIA indicates more age dependence within this range (see **Figure 6**). All assays work with either serum or plasma, though the former is preferred; whereas the TRFIA, like the Phoenix assay, measures directly in serum (68), and the LC–MS/MS assay requires prior sample extraction and processing (26, 114, 115). The TRFIA analysis shows that measuring in plasma underestimates INSL3 by approximately 10% compared to serum (68). Direct comparison of serum samples from men of different ethnicity shows no difference between Caucasian, South Asian, or Afro-Caribbean heritage (68). Recent population studies also suggest no difference between Chinese and other ethnicities (68). There are other less well-validated commercial assays available; however, caution is warranted since these may give unreliable and highly erroneous results (116). All of the findings reported in this review are based on population studies and animal research. There is currently no experience of using INSL3 as a clinical parameter on individual patients, though practical utility studies are planned and essential for establishing INSL3 as a useful addition to the andrological armamentarium.

**Figure 6:**
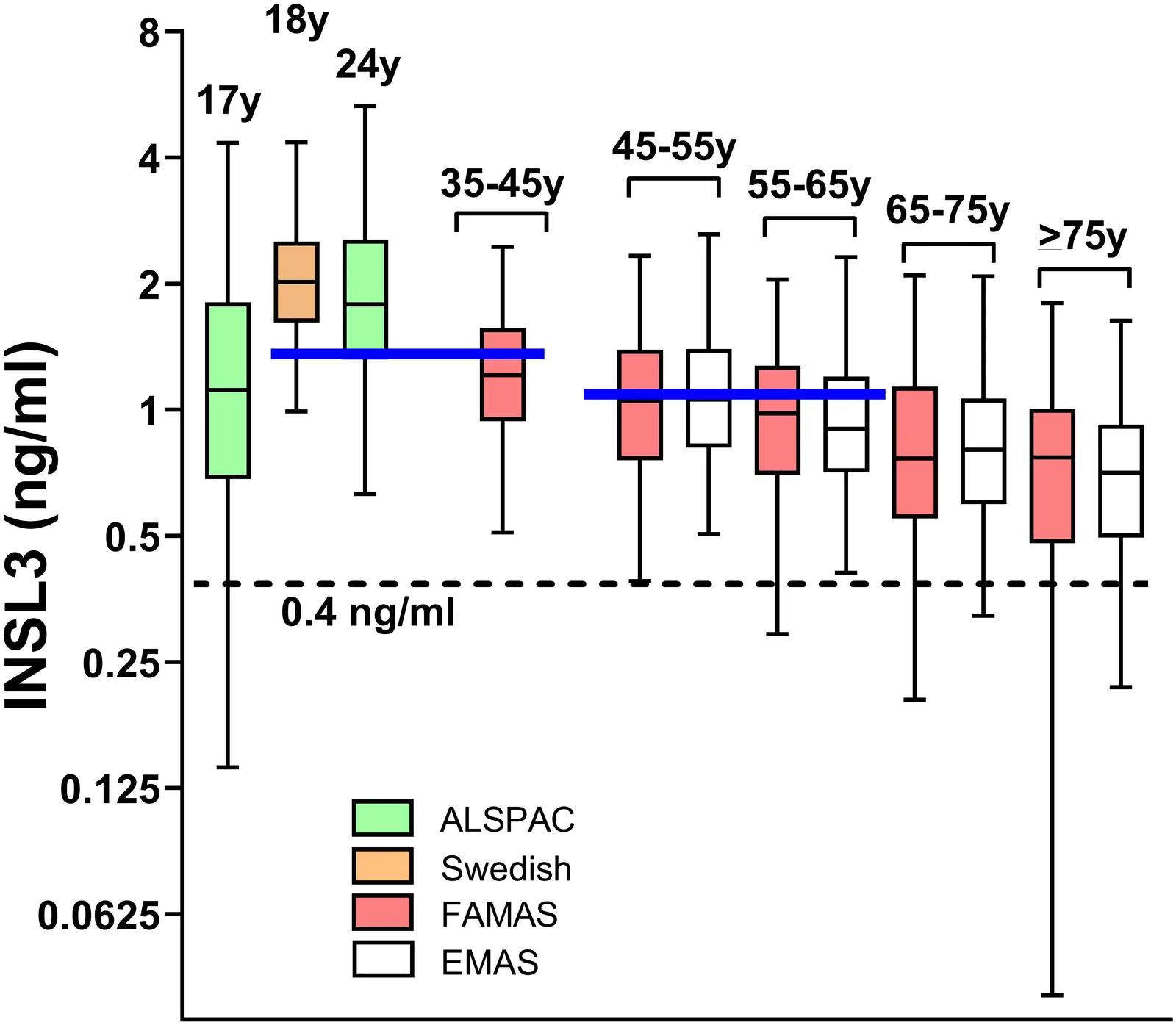
Means and 95% CI for circulating INSL3 in various age groups derived from the ALSPAC (green) (28), Swedish (orange) (64), FAMAS (red) (69), and EMAS (white) (67) cohorts, as indicated, all measured using the same TRFIA assay. Each age range is non-overlapping, i.e., 35-45y represents subjects aged from 35.0 to 44.9 years, etc. The horizontal black dashed line represents the 0.4-ng/mL threshold, also representing the lower reference limit (-2SD) for this cohort of ca. 1,200 men, below which it is hypothesized that hypogonadism in older men is indicated (68). The horizontal blue lines indicate the means assessed by using the LC–MS/MS method for a similarly aged Danish cohort (26). INSL3 concentration is represented as a log_2_ scale [modified after (68)].

The same applies to routine applications in animal studies. The potential applications of INSL3 assessment are listed in **Table 1** for both the human clinical as well as animal sectors. Well-validated INSL3 TRFIA assays have been described for ruminants and pigs (66), dogs (90), and rodents (16).

**Table 1:** Potential applications of male INSL3 testing in clinical and other contexts.

Life phase	Clinical application	Alternative species application
Fetus	Monitoring mid-pregnancy amniotic fluid for sex-appropriate development.	Monitoring male fetal development, regarding the effects of EDCs, pharmaceuticals, etc. Fetal sex determination.
Cord-blood and/or minipuberty	Monitoring for sex-appropriate development.	Monitoring for sex-appropriate development.
Puberty	Biochemical Tanner staging to monitor pubertal development. Assessment of CHH, CDGP, Klinefelter, and their treatments.	Biochemical staging to monitor pubertal development.
Adult	Simple assessment of functional hypogonadism (INSL3 <0.4 ng/mL). Monitoring the progression of illness and recovery (e.g., during COVID). Assessing the impact of anabolic steroid misuse and/or iatrogenic hypogonadism caused by treatment. Monitoring the impact and recovery from steroidal or HPG-suppressive contraceptive regimens.	Assessment of hypogonadism caused by reprotoxic environmental exposure or other stressors (e.g., heat). Assessment of Leydig cell capacity in pharmaceutical and toxicology testing. Anti-doping testing for anabolic steroid misuse (e.g., in horses, camels). Monitoring the impact and recovery from steroidal or HPG-suppressive contraceptive regimens.

Other than for assessment of functional hypogonadism in aging men (68), thresholds and reference limits for the normal population still need to be determined. Because of the species specificity of INSL3 assays, applications in non-human species still require rigorous validation.

## Summary and conclusions

The HPG axis in the male represents an integrated endocrine system connecting the brain via the hypothalamus to the anterior pituitary and thence to the testes, where both steroidogenic and spermatogenic compartments are more or less separately targeted. Homeostatic regulation is achieved by negative feedback on the one hand by testicular androgens, representing the steroidogenic compartment (also converted to estrogens), acting in the brain, and on the other hand by inhibin B, representing the seminiferous compartment, to the anterior pituitary. Measuring any of these individual components reflects not only the functioning of the tissue and cells from which they originate but also the dynamic status of the HPG axis as a whole. All of these are in a state of acute homeostatic regulation, with GnRH, the pituitary gonadotropins, and testosterone all pulsatile or phasic in their expression and with both amplitude and frequency modulation important in determining informational content (2). Moreover, each of these can be modulated extrinsically by other endocrine and metabolic factors acting at different sites within the HPG axis (117). These factors together contribute to the biological variance in each parameter when measured in the circulation, and consequently these parameters, though valuable, are less informative regarding the causality of measured changes. INSL3 is unusual in being constitutively expressed once Leydig cells have achieved their adult end-differentiation status and is not expressed significantly by any other cell type in the body. Hence, circulating INSL3 is the only parameter to provide definitive information regarding the steroidogenic component of the HPG axis target tissue, i.e., whether an individual is primarily hypogonadal and not where low testosterone is caused by some other influence elsewhere on the HPG axis. While acutely circulating INSL3 offers little biological variance, it can be chronically influenced by factors which affect either the number and/or differentiation state of Leydig cells. Chronic suppression of the HPG axis and, hence, LH drive can lead to Leydig cell dedifferentiation, as seen in systemic inflammation, in steroidal male contraception, in anabolic steroid misuse, or in seasonally breeding animals. Some viral illnesses appear directly to cause Leydig cell death, such as HIV (118), and possibly SARS-Cov-2 (119, 120), as well as chickenpox (65). Both situations lead to a reduction in circulating INSL3. Importantly, recent evidence suggests that Leydig cell loss or dedifferentiation may be reversible, presumably once the LH drive has been restored, and this can be assessed by measuring INSL3 (87, 95), though whether full restoration of gonadal status is possible may depend on the age of the individual and presumably the quantity and quality of Leydig cell precursors and/or stem cells.
